# Facile Preparation of a Novel HfC Aerogel with Low Thermal Conductivity and Excellent Mechanical Properties

**DOI:** 10.3390/gels9100839

**Published:** 2023-10-23

**Authors:** Wei Wang, Zhanwu Wu, Shicong Song, Qi You, Sheng Cui, Weimin Shen, Guoqing Wang, Xuanfeng Zhang, Xiaofei Zhu

**Affiliations:** 1 College of Materials Science and Engineering, Nanjing Tech University, Nanjing 210009, China; 2 Jiangsu Collaborative Innovation Center for Advanced Inorganic Function Composites, Nanjing Tech University, Nanjing 211800, China; 3 Shanghai Space Propulsion Technology Research Institute, Huzhou 313000, Chinassc18235926020@163.com (S.S.);

**Keywords:** HfC aerogel, sol-gel, carbothermal reduction, thermal insulation, compressive strength

## Abstract

Aerogels emerge as captivating contenders within the realm of high-temperature thermal resistance and thermal insulation. Nevertheless, their practical applications are usually constrained by their inherent brittleness when subjected to rigorous conditions. Herein, employing hafnium dichloride oxide octahydrate (HfOCl_2_·8H_2_O) as the hafnium source and resorcinol–formaldehyde (RF) as the carbon precursor, hafnium carbide (HfC) aerogels are fabricated via the sol-gel method complemented with carbothermal reduction reaction. Investigations are conducted into the effects of various molar ratios, duration, and temperatures of calcination on the microstructural features and physico-chemical characteristics of the as-prepared HfC aerogel. The aerogel shows a high BET-specific surface area (601.02 m^2^/g), which is much larger than those of previously reported aerogels. Furthermore, the HfC aerogel exhibits a low thermal conductivity of 0.053 W/(m·K) and a compressive strength of up to 6.12 MPa after carbothermal reduction at 1500 °C. These excellent thermal insulation and mechanical properties ensure it is ideal for the utilization of high-temperature thermal resistance and thermal insulation in the fields of aerospace.

## 1. Introduction

Aerogel, a type of nano-porous substance, is made of colloidal particles or polymer molecular chains that have a clear three-dimensional network skeletal structure. Aerogels are characterized by high specific surface area, low thermal conductivity, ultra-low density, and high porosity, along with other outstanding textural characteristics, etc. Aerogel has found extensive uses in many fields, such as thermal insulation [1,2], adsorption [3], catalysis [4], biological and medical applications [5], and energy storage and transformation [6]. Until now, oxide aerogels, such as SiO_2_, Al_2_O_3_, ZrO_2_, and TiO_2_ [7,8,9,10], have consistently demonstrated outstanding thermal stability and exceptionally low thermal conductivity in the air atmosphere. However, due to the multifaceted applications of aerogels, the inherent instability of SiO_2_ aerogels, particularly their susceptibility to structural collapse and densification at temperatures exceeding 800 °C, has posed a formidable challenge. This phenomenon considerably restricts the extensive utility of oxide aerogels in demanding high-temperature environments, a challenge of growing significance in various industries and scientific domains.

Carbide aerogels [11] exhibit excellent characteristics, including low density, high porosity, and exceptional resistance to high temperatures. These properties are complemented by the inherent attributes of carbides, such as high hardness, a high melting point, and chemical stability. This unique combination of features positions carbide aerogels as an innovative solution that holds great promise for overcoming the limitations typically associated with SiO_2_ aerogels within the realm of high-temperature thermal protection systems. Silicon carbide (SiC) aerogels were among the first carbide aerogels to be explored following the work of Lu et al. [12], who reported the successful synthesis of SiC/silica nanocomposites via the carbothermal reduction in composite SiO_2_ aerogels containing graphitic carbon nanofibers. This transformation took place within an argon atmosphere at 1650 °C, although no detailed properties of the resulting samples were provided. Leventis et al. [13] were the first to propose a method for the production of monolithic porous SiC aerogels, utilizing a polyacrylonitrile crosslinked SiO_2_ aerogel as a precursor. However, it is worth noting that their approach was marred by its intricate and cumbersome process, resulting in limited practicality. Unfortunately, they did not provide information regarding the insulating properties of these aerogels.

To investigate the thermal insulation capabilities of SiC aerogels, Kong et al. [11,14] proposed a novel method of preparing bulk SiC aerogel, making RF/SiO_2_ composite aerogel the precursor. The composite aerogel material exhibits a thermal conductivity of 0.026 W/(m·K), surpassing that of traditional carbon aerogels, all the while preserving the aerogel’s compressive strength. In pursuit of fortifying aerogels for deployment in the most demanding conditions, An et al. [15] prepared bulk SiC aerogel via the utilization of CF/SiO_2_ aerogel as a precursor. The aerogels exhibit impressive mechanical integrity, boasting a compressive strength of 1.32 MPa, in addition to a thermal conductivity of 0.049 W/(m·K) and a specific surface area of 162 m^2^/g. These qualities make them exceedingly suitable for applications in the aerospace sector. In addition to SiC aerogels, researchers have explored zirconium carbide (ZrC) as a promising material for aerogel fabrication due to its remarkable hardness, high melting point, and exceptional chemical stability. Ye et al. [16] produced a ZrCO/C composite aerogel, which exhibited an impressive specific surface area of 589 m^2^/g. Their findings revealed that the presence of ZrC crystals within the material acted as antioxidants, significantly enhancing the structural integrity of the aerogel. A novel kind of ternary ZrCO (C/ZrO_2_/ZrC) aerogel material possessing a specific surface area of 637.4 m^2^/g was fabricated by Cui et al. [17]. At ambient temperature, carbon fiber felt composite samples displayed an outstandingly low thermal conductivity of 0.057 W/(m·K). However, information regarding the compressive strength of these aerogels was not provided. Ren et al. [18] detailed the production of ZrC/C aerogels via the copolymerization of sols combined with the carbothermal reduction method, utilizing ZrOC and phenol formaldehyde (PF) sols, along with hexamethylene tetraamine (HMTA) as a crosslinker. The bulk density of ZrC/C aerogel was found to be 0.262 g/cm^3^ and the highest compressive strength was observed to be 4.42 MPa, whereas the thermal conductivity was 0.0896 W/(m·K). Collectively, previous reports indicate that the previously investigated aerogels have struggled to achieve a balance between thermal insulation properties and robust mechanical performance.

HfC, with an extraordinary melting point as high as 3890 °C, has the highest melting point among known single compounds. With a typical NaCl-type face-centered cubic crystal, HfC has advantages including but not limited to high specific strength, high hardness (Vickers hardness: 26l GPa), excellent chemical stability and high modulus of elasticity (350–510 GPa) owing to the coexistence of metal bonds and covalent bonds. Consequently, HfC has been extensively employed in various fields, for instance, in aerospace, industrial carbide, and coating applications [19,20,21], all of which involve harsh environments.

Matović et al. [22] conducted the synthesis of HfC utilizing the sol-gel polycondensation method, employing hafnium chloride and citric acid monohydrate as the primary source materials. The formation of HfC initiated at a temperature of 1000 °C, culminating in the production of nearly pure HfC (∼97%) when subjected to a temperature of 1600 °C. In another significant study, Kim et al. [23] reported the successful synthesis of HfC via high-temperature heat treatment involving exposure to 1700 °C. In their approach, HfO_2_ served as the hafnium source, while carbonized phenolic resin was utilized as the carbon precursor. Their results underscored the significance of maintaining a specific molar ratio of C/Hf, notably 3.3, for achieving the highest purity level. Additionally, Feng et al. [24] successfully manufactured ultrafine HfC powders by employing a combination of the carbothermal reduction method and the discharge plasma sintering technique. While the synthesis of HfC has been the subject of numerous studies, it is noteworthy that the majority of the synthesized materials have primarily taken the form of powders and nanoparticles. Porous HfC structures, on the other hand, have remained relatively underreported within the existing body of literature.

Accordingly, this manuscript has taken into account the molar ratio of reactants, as well as the heat treatment time and temperature discussed in the aforementioned studies, while capitalizing on HfC’s high-temperature resistance and exceptional mechanical properties, aiming to prepare HfC aerogel materials with a three-dimensional network structure, thereby enriching the range of aerogel materials and addressing applications in extreme harsh environments.

Hence, with the help of the extensive prior research on carbide aerogels and HfC synthesis by predecessors, we tried to present a novel method that combines the exceptional physicochemical attributes of HfC with the lightweight and efficient thermal insulation of aerogels, resulting in the creation of innovative HfC aerogels. This material holds the potential to seamlessly unite thermal insulation properties with remarkable mechanical characteristics. In the present study, HfC aerogel was fabricated by mixing HfO_2_ sol and RF mixed solution in a beaker, followed by the sol-gel method, CO_2_ supercritical fluid drying technique, and carbothermal reduction process under an inert atmosphere using argon or helium. In addition, we have a comprehensive examination of the influence of different Hf/R molar ratios, heat treatment temperatures, and holding times on the microstructures and properties of the aerogels. Finally, the HfC aerogels demonstrated impressive characteristics, including low density, exceptional heat resistance, high compressive strength, and low thermal conductivity.

## 2. Results and Discussion

### 2.1. Thermal Conductivity and Compressive Strength Analysis

Table 1 presents the density, compressive strength, and thermal conductivity of the aerogels subjected to heat treatment at 1500 °C. As indicated in Table 1, the bulk densities of the aerogels exhibit a decreasing trend with increasing Hf/R molar ratios, with the HfC aerogel achieving an impressively low bulk density of 0.37 g/cm^3^ (C6). The resultant aerogels exhibit significant promise for deployment in challenging environments, thanks to the enhanced mechanical strength attained via the carbothermal reduction process, surpassing that of oxide aerogels. Notably, the compressive strength of the HfC aerogel was measured at a remarkable 6.12 MPa, a significant improvement over the previously reported 4.42 MPa for ZrC aerogels, as documented by Ren [18]. The HfC aerogel also boasts an exceptional thermal conductivity of 0.053 W/(m·K) at 25 °C, demonstrating a substantial advantage over the 0.057 W/(m·K) reported by Cui [17]. Furthermore, an infrared photograph (Figure S4) depicts the results of a 200 °C thermal insulation evaluation conducted on a carbon fiber mat composite HfC aerogel; over the course of time, aerogel still exhibits excellent thermal insulation characteristics.

### 2.2. Synthetic Route and Reaction Mechanism Analysis

**Figure 1:**
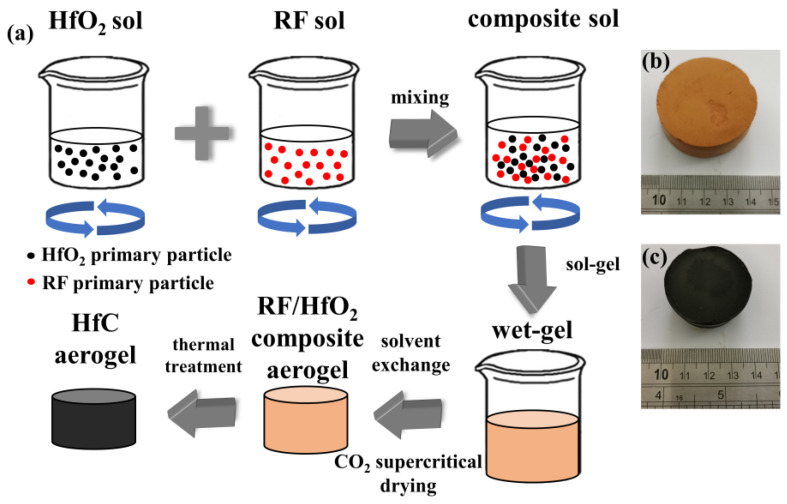
(**a**) Process flow chart of HfC aerogel preparation, the macro sample diagram of (**b**) RF/HfO_2_ composite aerogel, and (**c**) HfC aerogel.

As shown in Figure 1, HfOCl_2_·8H_2_O and RF were separately dissolved in water and ethanol and agitated before being thoroughly stirred for a specified duration. Subsequently, they were combined and stirred to create an RF/HfO_2_ composite sol. The RF/HfO_2_ composite aerogel was obtained via a CO_2_ supercritical process, and finally, HfC aerogel was obtained after high-temperature heat treatment. As a result of the pyrolysis of organic components, the HfC aerogels underwent linear shrinkages during the carbonization process [12], as illustrated in Figure 1b,c. In addition, carbide aerogels are frequently utilized in conjunction with fiber mats to enhance additional stretchability and flexibility.

**Figure 2:**
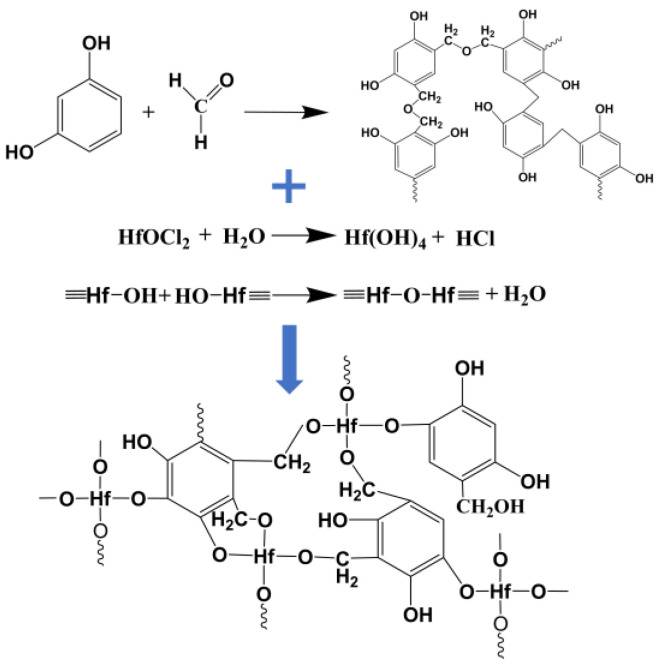
Reaction mechanism diagram of RF/HfO_2_ composite wet gel in sol-gel process.

Figure 2 illustrates the formation of the RF/HfO_2_ composite wet gel via a series of condensation and crosslinking reactions. Initially, hydroxymethyl groups (–CH_2_OH) originating from aldehyde and hydroxyl groups engage in addition reactions, leading to the incorporation of hydroxymethyl resorcinol and –OH from hafnium oxychloride hydrolysates. Subsequently, these functional groups undergo condensation, resulting in the formation of methylene (–CH_2_–), methylene-ether (–CH_2_OCH_2_–), Hf–O–Hf, and Hf–O–C bridges. The process culminates in the completion of the RF/HfO_2_ composite wet gel via crosslinking and agglomeration. Table 2 reveals an interesting trend: as the molar ratio of Hf/R increases, the gelation time decreases. This phenomenon can be attributed to the heightened probability of collision nucleation between particles within the sol-gel system as the Hf concentration rises. Increased Hf concentration leads to a decrease in pH, creating a more acidic environment that enhances the sol-gel process of RF and subsequently results in shorter gelation times for the samples.

### 2.3. Chemical Composition and Structural Analysis

**Figure 3:**
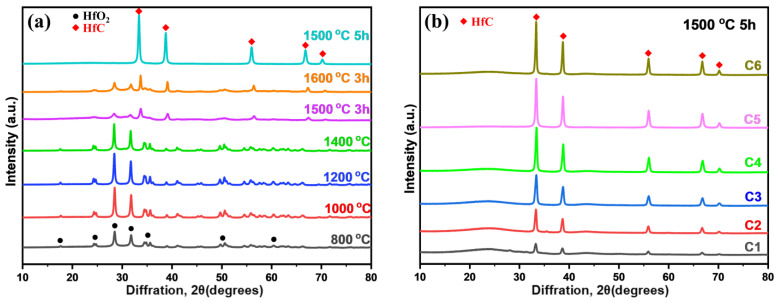
(**a**) XRD patterns of the HfC aerogel heat-treated at various temperatures and for various time durations (800 °C, 1000 °C, 1200 °C, 1400 °C, 1500 °C, and 1600 °C for 3 h; 1500 °C for 5 h), (**b**) XRD patterns of the HfC aerogel with various Hf/R molar ratios heat-treated at 1500 °C for 5 h.

Figure 3 shows the XRD diffractograms of the HfC aerogels subjected to various calcination temperatures ranging from 800 to 1600 °C. Initially, when heat-treated at 800 °C for 3 h, distinct peaks at 24.64°, 28.30°, 31.76°, and 50.46° corresponding to the (0 1 1), (−1 1 1), (1 1 1), and (0 2 2) planes of the t-HfO_2_ phase (JCPDS: No.53-0550) begin to emerge in the aerogels. Notably, heat treatment at 1000 °C and 1200 °C results in sharper t-HfO_2_ diffraction peaks. However, upon exposure to the elevated temperature of 1400 °C for 3 h, the peak intensities corresponding to the t-HfO_2_ phases decrease. This observation may be indicative of the initiation of carbon-thermal reduction processes, albeit with some uncertainty regarding the trace presence of HfC. Upon subjecting the aerogels to heat treatment at 1500 °C for 3 h, the peak intensities associated with HfO_2_ phases substantially decrease when compared to the 1400 °C sample. In this case, diffraction peaks at 33.4°, 38.8°, 56.1°, 66.8°, and 70.1° emerge, attributed to the (1 1 1), (2 0 0), (2 2 0), (3 1 1), and (2 2 2) planes of the HfC phase (JCPDS: No.65-0964). As the aerogel undergoes further heat treatment at 1600 °C for 3 h, the peak intensities associated with the HfC phase continue to rise, indicating the ongoing extent of carbon-thermal reduction at higher temperatures. However, it is worth noting that the HfO_2_ peaks still persist. Remarkably, after carbothermal reduction at 1500 °C for 5 h, the XRD pattern reveals only peaks corresponding to the HfC phase. This implies a complete reaction between HfO_2_ nanoparticles and the amorphous carbon present in the aerogel. Figure 3b highlights a noteworthy trend, wherein the prominence of the HfC phase increases with higher Hf/R molar ratios.

**Figure 4:**
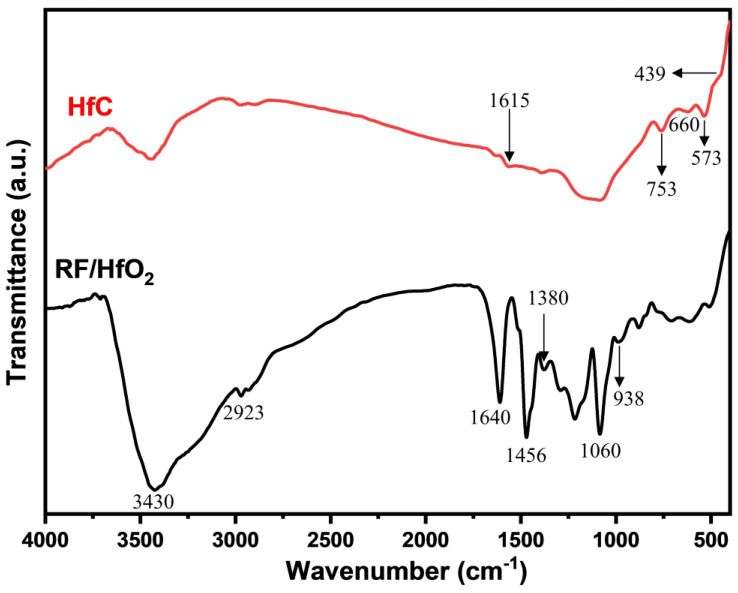
FT−IR spectra of the RF/HfO_2_ and HfC aerogels.

In Figure 4, the FT−IR spectra of RF/HfO_2_ and HfC aerogels fabricated under reaction conditions are displayed. The broad absorption band at 3430 cm^−1^ is attributed to the –OH bonds within water molecules adsorbed within the sample [25,26]. At 2923 cm^−1^, the spectrum reveals the stretching vibration absorption of the C−H bond found in –CH_2_. The C–O bond is associated with the bands at 1240 cm^−1^ and 1060 cm^−1^. Notably, the formation of the benzene skeleton within the RF system gives rise to the distinctive band observed at 1456 cm^−1^ [27]. Further spectral features emerge in the region of 1640 cm^−1^ and 1615 cm^−1^, corresponding to the stretching vibration of the C = C bond. The presence of Hf–O bonds is evidenced by bands at 439 cm^−1^, 660 cm^−1^, and 753 cm^−1^ [28].

**Figure 5:**
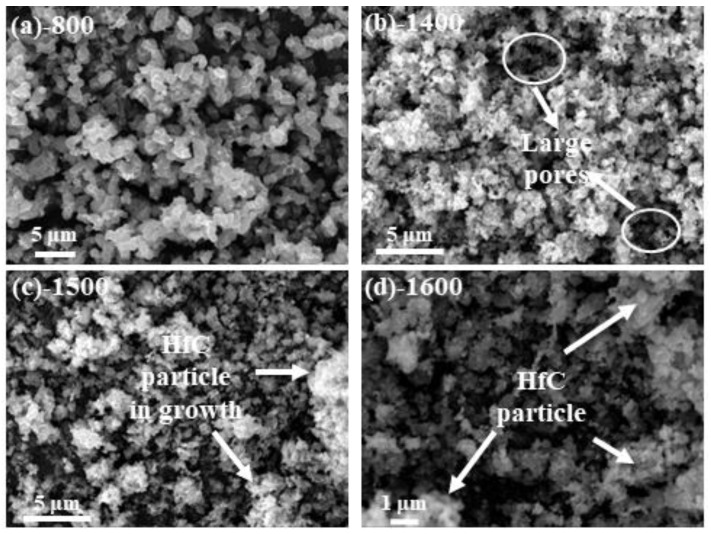
SEM micrographs of the aerogels heat-treated at various temperatures under a flowing argon atmosphere: (**a**) 800 °C, (**b**) 1400 °C, (**c**) 1500 °C, and (**d**) 1600 °C.

In Figure 5, the microstructures of HfC aerogels following thermal processing under a flowing argon environment at various temperatures are depicted. The unique characteristics of these aerogels are a result of the distinct hydrolysis and condensation rates of HfO_2_ and RF sols. As a consequence, the sample exhibits a disordered yet uniformly distributed network structure of nanoparticles after heat treatment. The composite aerogel is fabricated by combining carbon and HfO_2_ nanoparticles at 800 °C for 3 h, as illustrated in Figure 5a. In this micrograph, the sample displays a chaotic yet compact porous structure interspersed with numerous micropores. The HfO_2_ nanoparticles are encapsulated by carbon particles. With carbothermal reduction at 1400 °C for 3 h, the particle size decreases, resulting in a more homogeneous aerogel structure with significantly fewer large pores, as depicted in Figure 5b. Moving to Figure 5c, it becomes evident that the carbothermal reduction process commences as the temperature is raised to 1500 °C for 3 h, signaling the interaction between amorphous carbon and HfO_2_. Finally, Figure 5d illustrates the outcome after 3 h of calcination at 1600 °C, where the HfC particle population has substantially increased, and the nanoparticles are in close proximity to one another. This is attributed to the consumption of HfO_2_ as it undergoes reactions with carbon particles.

**Figure 6:**
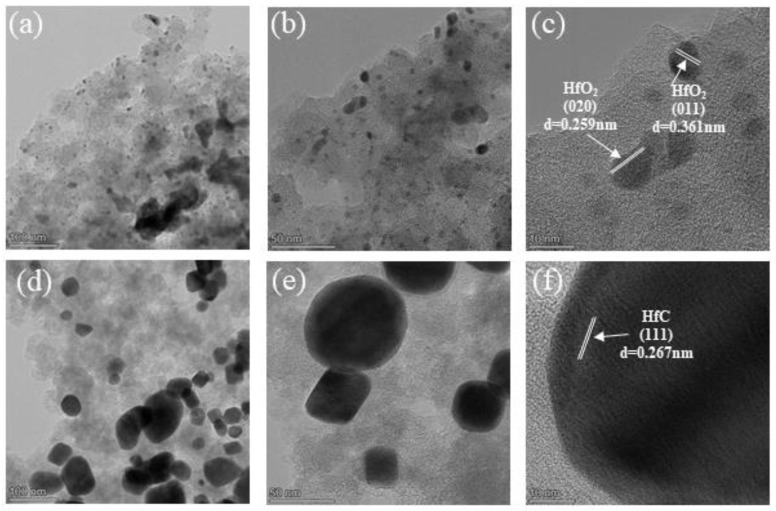
TEM images of the samples heat−treated at various temperatures under a flowing argon atmosphere: (**a**–**c**) 800 °C; (**d**–**f**) 1500 °C.

Figure 6a–c provide compelling visual evidence of the presence of HfO_2_ particles within the aerogels. Notably, the distinct lattice fringes with interplanar distances measuring 0.259 nm and 0.361 nm correspond to the (020) and (011) planes of HfO_2_, as they appear in Figure 6c. These HfO_2_ structures are intimately surrounded by carbon nanoparticles, offering insight into the composite’s microstructure. Figure 6d shows the emergence of HfC particles within the composite aerogels following heat treatment at 1500 °C for 3 h. The HfC particles exhibit diameters in the approximate range of 50–100 nm, corroborating findings from SEM observations depicted in Figure 6d. Within this micrograph, a distinct lattice fringe with an interplanar distance of 0.267 nm is observable, attributing this feature to the (111) plane of HfC. This finding further validates the premise that carbothermal reduction leads to the transformation of HfO_2_ into HfC particles. It is noteworthy that in Figure 6d,e, a trace amount of HfO_2_ is still discernible, which is in alignment with the XRD analysis.

### 2.4. Thermogravimetric and Pore Morphology Analysis

**Figure 7:**
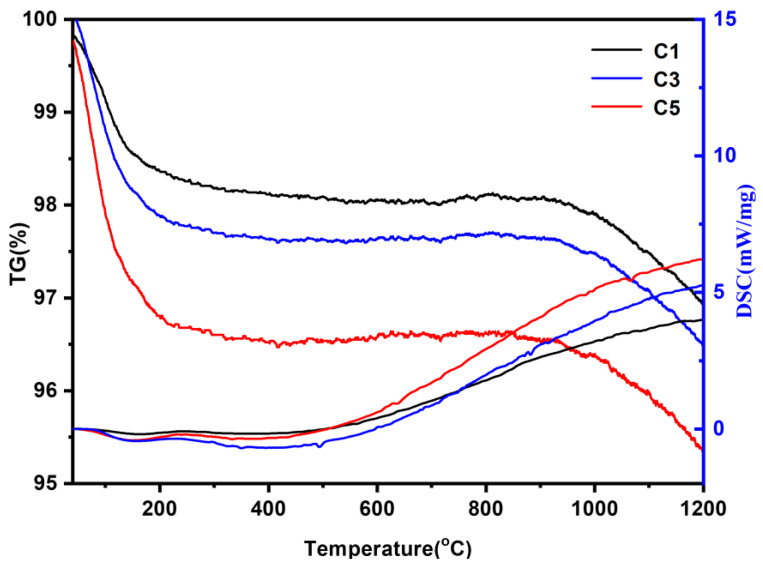
TG and DSC curves of the HfC aerogels with different Hf/R molar ratios under flowing argon.

The TG and DSC curves of HfC aerogels with various Hf/R molar ratios obtained under an argon atmosphere are shown in Figure 7. TGA-DSC analysis serves as a valuable tool for assessing the sample′s thermal stability. The thermogram profile can be delineated into three distinct sections, as depicted in Figure 7: (1) 30–200 °C, (2) 200–900 °C, and (3) 900–1200 °C. In the initial stage (30–200 °C), a minor weight loss of approximately 1.2–3% is observed. This initial weight loss primarily results from the evaporation of ethanol and the removal of physically adsorbed water. The subsequent stage, spanning the temperature range of 200–900 °C, exhibits a gradual mass loss of approximately 0.5%. The third weight loss stage, occurring within the temperature range of 900–1200 °C, exhibits a mass loss of approximately 1.3%. This phenomenon can be attributed to the reaction between the remaining unreacted oxides within the HfC aerogel and carbon aerogel particles, leading to a continued reaction at elevated temperatures and the generation of HfC. This process results in a further mass loss within the aerogel. Notably, as the molar ratio of Hf/R increases, there is an escalation in mass loss at high temperatures, as evident from the figure. This observation underscores the remarkable temperature resistance exhibited by HfC aerogel within an inert atmosphere.

**Figure 8:**
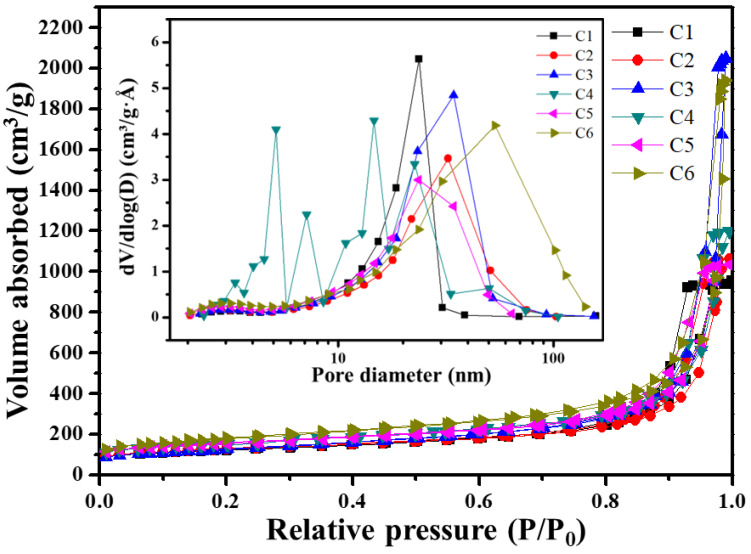
Nitrogen sorption isotherms and pore size distribution of the aerogels heat-treated at 1500 °C.

Figure 8 presents nitrogen sorption isotherms classified of the HfC aerogels according to the IUPAC standards. The curves depicted in Figure 8 are characteristic of type-IV isotherms with H1-type hysteresis loops, a hallmark of mesoporous materials featuring cylindrical pores. The adsorption ratio (P/P0) of less than 0.05 is indicative of micropores dominating the structure. In the vicinity of relative pressures around 0.5, both mesopores and macropores exhibit multi-layer adsorption processes on their external surfaces. The absence of an adsorption plateau as P/P0 approaches suggests that only a limited number of macropores are present. Table 3 provides the average pore sizes, typically falling within the range of 10–23 nm for this mesoporous material. Furthermore, it is noteworthy that the specific surface areas exhibit an increasing trend as the Hf/R molar ratio increases. For instance, sample C6, with an Hf/R ratio of 2:1, boasts an average pore diameter of 23.34 nm and a maximum surface area of 601.02 m^2^/g. This is notably higher than those reported in previous studies on carbide aerogels [14,15,16,29].

## 3. Conclusions

In this study, we introduce an innovative methodology that combines HfC, renowned for its outstanding physico-chemical properties, with a lightweight and exceptionally efficient thermal insulating aerogel to create HfC aerogels. The aerogel sample was fabricated via the sol-gel method, complemented with carbothermal reduction reaction under flowing argon. The aerogel exhibits an impressive compressive strength of 6.12 MPa, a significant enhancement compared to previously reported carbide aerogels. This exceptional strength overcomes the inherent fragility often associated with aerogels, making it a reliable choice for demanding applications. In addition, the specific surface area of the aerogel reaches a remarkable 601.02 m^2^/g, a value surpassing those found in previously reported literature on carbide aerogels. In the case of the carbon fiber mat composite aerogel, the thermal conductivity is as low as 0.053 W/(m·K) at ambient temperature. These remarkable thermal insulation and mechanical properties position it as an ideal candidate for applications requiring high-temperature thermal resistance and thermal insulation, particularly within the aerospace industry.

## 4. Materials and Methods

### 4.1. Materials

HfOCl_2_·8H_2_O was purchased from Alfa Aesar Co., Ltd., Shanghai, China. Citric acid and resorcinol were purchased from Xilong Chemical Co., Ltd., Guangzhou, China. Formaldehyde was purchased from Shanghai Lingfeng Chemical Reagent Co., Ltd., Shanghai, China. Absolute ethyl alcohol and deionized water were provided by Wuxi City Yasheng Chemical Co., Ltd., Wuxi, China, and Nanjing Wanqing Chemical Glassware & Instrument Co., Ltd., Nanjing, China., respectively.

### 4.2. Synthesis

In this study, the hafnium source was HfOCl_2_·8H_2_O, the carbon sources were RF, and the crosslinking agent was CA. EtOH was utilized as the solvent, and deionized water served as the hydrolysis agent. No further purification was conducted on any of the reactants or solvents before use, and no catalyst was used in the experiment.

The following procedures were used to prepare the aerogel. HfO_2_ precursor sol formation: In the initial step, a HfO_2_ precursor sol was created by combining HfOCl_2_·8H_2_O, citric acid (CA), and deionized water in a molar ratio of 1:0.1:50. The mixture was stirred at 50 °C for 1 h. RF mixed solution preparation: Subsequently, an RF mixed solution was prepared by combining resorcinol (R), formaldehyde (F), and ethanol (EtOH) in a molar ratio of 1:2:50. This solution was stirred at room temperature for approximately 30 min. Combining RF and HfO_2_ solutions: the RF solution and HfO_2_ precursor sol were then combined in a container and stirred for approximately 30 min at room temperature. Gel formation: the resulting mixture was transferred into plastic molds and allowed to gel for 1 h at 50 °C. Aging: the wet gels were subjected to aging at 50 °C for one day to facilitate the exchange of reaction byproducts and water from the material′s pores. Solvent exchange: subsequently, the gel components were immersed in an absolute ethanol bath for a duration of 5 days. Supercritical drying: To produce RF/HfO_2_ composite aerogels, CO_2_ supercritical fluid drying was employed. The temperature was gradually increased to 50 °C while maintaining a pressure of 100 bar. Carbothermal reduction: the red-yellow RF/HfO_2_ composite aerogels underwent carbothermal reduction for 5 h at various heat-treatment temperatures, resulting in the formation of black HfC aerogels.

The high-temperature heat treatment was carried out using the GSL-1750-KS model, a single-temperature-zone atmosphere tube furnace capable of reaching temperatures up to 1750 °C. This furnace is manufactured by Hefei Kejing Material Technology Co., Ltd. To ensure precise control over the temperature, a gradual ramping scheme was employed, with the temperature increasing at a rate of 2 °C per minute. The heat treatment process was divided into two distinct stages. The initial phase involved carbonization, conducted at a temperature of 800 °C, and this phase lasted for a duration of 3 h. Following the carbonization phase, the subsequent stage comprised the carbon thermal reduction process, extending over a period of 3 to 5 h. Notably, this process was carried out in an atmosphere of helium (He), with a continuous gas flow maintained at a rate of 100–150 mL/min throughout the procedure.

### 4.3. Characterizations

Thermal gravimetric analysis (TGA) and differential scanning calorimetry (DSC) were performed on a German NETZSCH STA449 F3 thermogravimetric analyzer (Selb, Germany) with a heating rate of 10 °C/min. X-ray diffraction (XRD) data were obtained using a Japanese Rigaku Smartlab equipped with a 9 KW X-ray source and CuKα1 radiation (λ = 0.15406 nm). Scanning electron microscopy (SEM) was conducted with an American Phenom Pharos G2 field emission scanning electron microscope (Kingston, PA, USA). Surface area, pore distribution, and pore volume measurements were carried out using a Micromeritics ASAP 2460 instrument (Norcross, GA, USA) which is manufactured by Micromeritics instrument Ltd. America. Infrared spectral analysis was performed using an American Thermo Scientific Nicolet iS20 infrared spectrometer (Waltham, MA, USA). Transmission Electron Microscopy (TEM) imaging was conducted with an American Thermo Scientific Talos F200X G2 (Waltham, MA, USA) operating at 200 kV. Compressive strength testing was conducted using a Shanghai INSTRON 3382 testing device (Norwood, MA, USA) on samples in the form of 30 mm cubic cubes. The testing was performed at a speed of 2.0 mm/min and at a temperature of 25 °C. To measure the thermal conductivity, carbon fiber mat-reinforced aerogel composites (40 mm × 40 mm × 10 mm) were degassed in a vacuum at 90 °C for 6 h and analyzed using a German Hot Disk-2500 thermal constant analyzer (Elk Grove, CA, USA).

## Figures and Tables

**Table 1:** The density, compressive strength, and thermal conductivity of HfC aerogel with various Hf/R molar ratios.

Sample	Bulk Density (g/cm^3^)	Compressive Strength (MPa)	Thermal Conductivity (W/(m·K))
C1	0.57	6.12 ± 0.13 SD	0.053 ± 0.003 SD
C2	0.50	5.22 ± 0.09 SD	0.055 ± 0.005 SD
C3	0.53	4.03 ± 0.19 SD	0.056 ± 0.005 SD
C4	0.49	3.45 ± 0.21 SD	0.059 ± 0.012 SD
C5	0.45	3.11 ± 0.07 SD	0.060 ± 0.009 SD
C6	0.37	2.45 ± 0.11 SD	0.062 ± 0.023 SD

**Table 2:** The gelation time of HfC aerogel precursors with various Hf/R molar ratios.

Sample	Hf/R Molar Ratio	Gelation Time (min)
C1	0.25:1	176
C2	0.5:1	158
C3	0.75:1	115
C4	1:1	81
C5	1.5:1	65
C6	2:1	46

**Table 3:** Pore structures of the specimens heat-treated at 1500 °C with various Hf/R molar ratios.

Sample	BET Surface Areas (m^2^/g)	Average Pore Diameters (nm)
C1	407.33	16.20
C2	420.66	19.23
C3	433.53	22.01
C4	514.29	10.95
C5	538.66	15.68
C6	601.02	23.34

## Data Availability

The raw/processed data required to reproduce these findings cannot be shared at this time as the data also form part of an ongoing study.

## Supplementary Materials

The following supporting information can be downloaded at https://www.mdpi.com/article/10.3390/gels9100839/s1. Figure S1. XRD patterns of the HfC aerogel with various Hf/R molar ratios, heat-treated at various temperatures and for various time durations. Figure S2. SEM images of the HfC aerogel heat treated at different temperatures (a–c) 800 °C; (d–f) 1400 °C; (g–i) 1500 °C; (j–l) 1600 °C. Figure S3. Mapping images of the HfC aerogel heat treated at different temperatures. Figure S4. Infrared thermal images of the HfC aerogel.
