# Supplementary material for: Facile Preparation of a Novel HfC Aerogel with Low Thermal Conductivity and Excellent Mechanical Properties
Source: Gels. 2023 Oct 23;9(10):839. doi: 10.3390/gels9100839 (PMC10606605; doi:10.3390/gels9100839)
Supplement: Supplementary file 1 [file gels-09-00839-s001.zip › gels-2648534-supplementary.pdf]

Supplementary Information

Facile Preparation of a Novel HfC Aerogel with Low Thermal Conductivity and Excellent Mechanical Properties

WeiWang [1,2,3], Zhanwu Wu [3], Shicong Song [3], Qi You [1,2], Sheng Cui [1,2,*], Weimin Shen [3,*], Guoqing Wang [3], Xuanfeng Zhang [3] and Xiaofei Zhu [3]

1 College of Materials Science and Engineering, Nanjing Tech University, Nanjing, 210009, China

2 Jiangsu Collaborative Innovation Center for Advanced Inorganic Function Composites, Nanjing Tech University, 211800 Nanjing, China

3 Shanghai Space Propulsion Technology Research Institute, 313000 Huzhou, China

* Correspondence: cui2002sheng@126.com (S.C.); sunhuisd@163.com (W.S.)

XRD patterns of the HfC aerogel

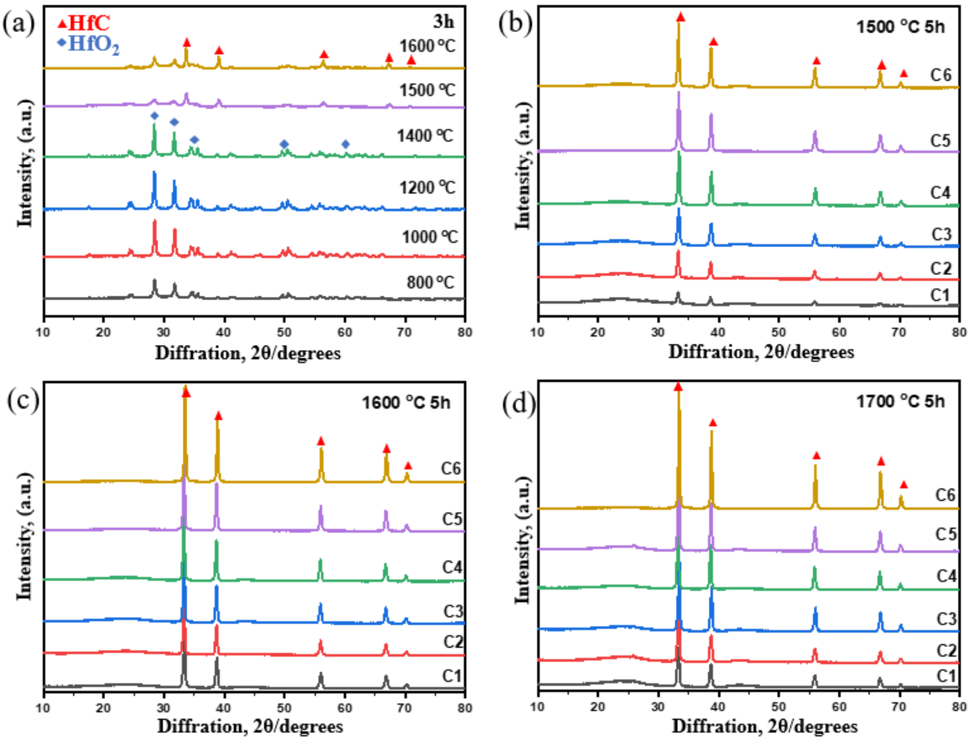

Figure S1. XRD patterns of the HfC aerogel with various Hf/R molar ratios, heat-treated at various temperatures and for various time durations.

SEM analyses of the HfC aerogel

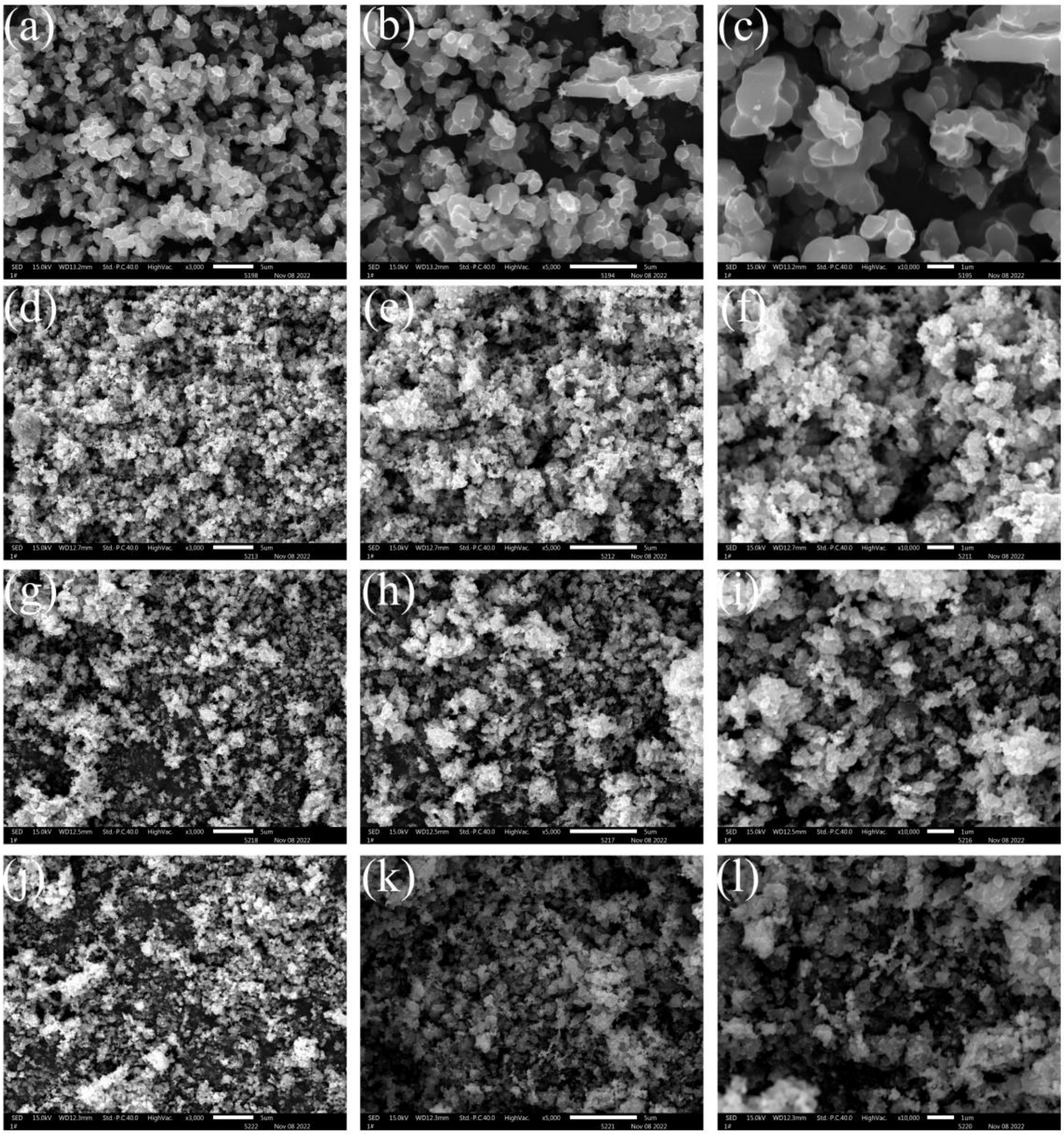

Figure S2. SEM images of the HfC aerogel heat treated at different temperatures (a-c) 800 °C; (d-f) 1400 °C; (g-i)1500 °C; (j-l) 1600 °C.

Mapping images of the HfC aerogel

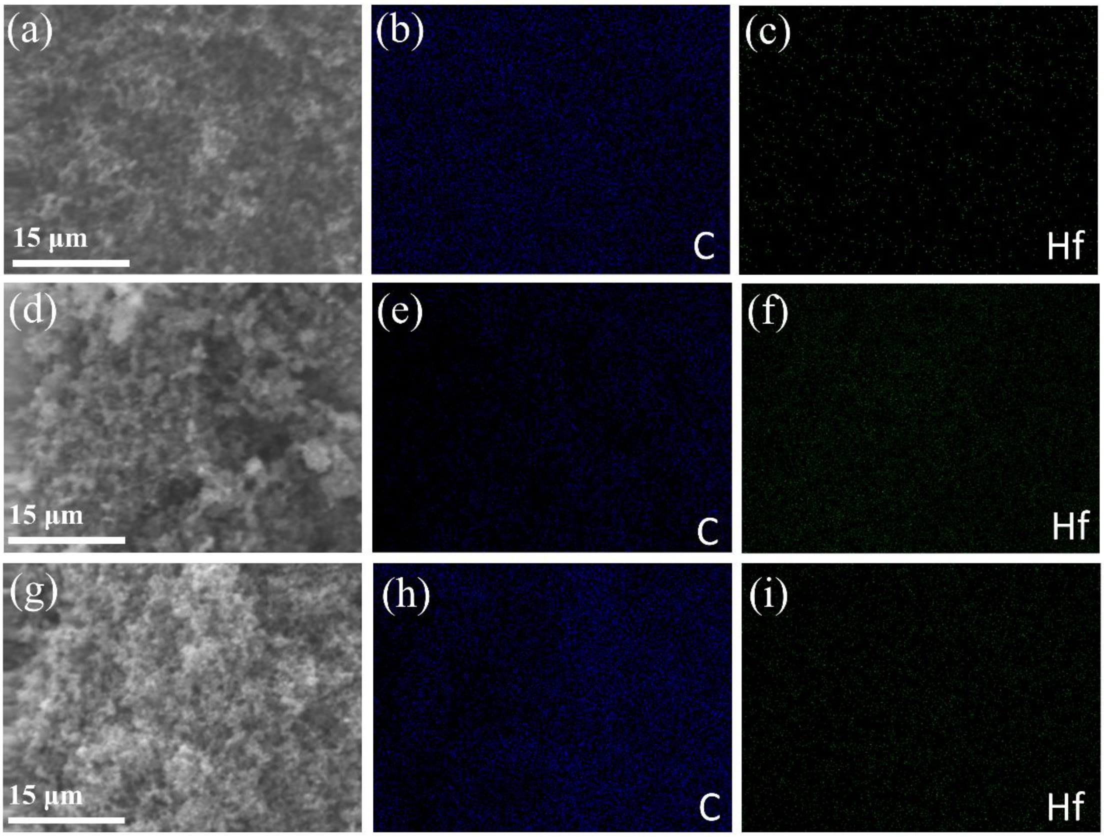

Figure S3. The elemental mapping of HfC aerogels at different heat treatment temperatures: (a-c) 1400 °C, (d-f)1500 °C and (g-i) 1600 °C

Infrared thermal images of the HfC aerogel

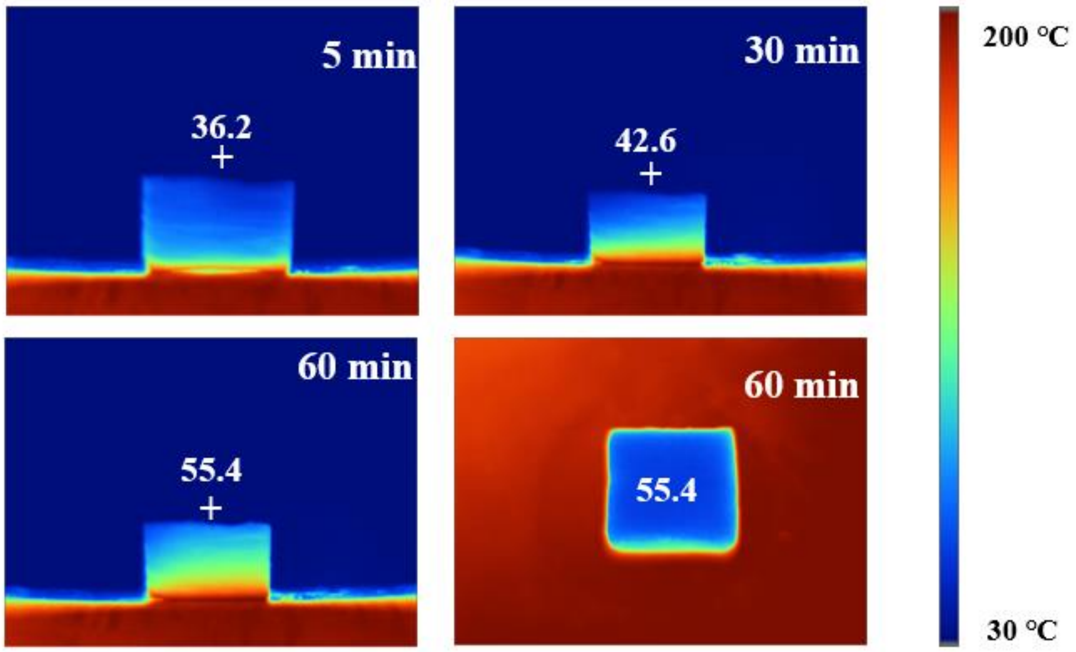

Figure S4. Infrared photo of 200 °C thermal insulation test of the carbon fiber mat composite HfC aerogel
